# Evaluation of Image Enhancement Method on Target Registration Using Cone Beam CT in Radiation Therapy

**DOI:** 10.4137/cmo.s512

**Published:** 2008-03-28

**Authors:** Hui Yan, Ren Lei, Jackie Wu, Fu Di, Fang-Fang Yin

**Keywords:** image-guided radiation therapy (IGRT), cone beam computed tomography (CBCT), image registration

## Abstract

An intensity based six-degree image registration algorithm between cone-beam CT (CBCT) and planning CT has been developed for image-guided radiation therapy (IGRT). CT images of an anthropomorphic chest phantom were acquired using conventional CT scanner and corresponding CBCT was reconstructed based on projection images acquired by an on-board imager (OBI). Both sets of images were initially registered to each other using attached fudicial markers to achieve a golden standard registration. Starting from this point, an offset was applied to one set of images, and the matching result was found by a gray-value based registration method. Finally, The registration error was evaluated by comparing the detected shifts with the known shift. Three window-level (WL) combinations commonly used for image enhancement were examined to investigate the effect of anatomical information of Bony only (B), Bone+Tissue (BT), and Bone+Tissue+Air (BTA) on the accuracy and robustness of gray-value based registration algorithm. Extensive tests were performed in searching for the attraction range of registration algorithm. The widest attraction range was achieved with the WL combination of BTA. The average attraction ranges of this combination were 73.3 mm and 81.6 degree in the translation and rotation dimensions, respectively, and the average registration errors were 0.15 mm and 0.32 degree. The WL combination of BT shows the secondary largest attraction ranges. The WL combination of B shows limited convergence property and its attraction range was the smallest among the three examined combinations (on average 33.3 mm and 25.0 degree). If two sets of 3D images in original size (512 × 512) were used, registration could be accomplished within 10~20 minutes by current algorithm, which is only acceptable for off-line reviewing purpose. As the size of image set reduced by a factor of 2~4, the registration time would be 2~4 minutes which is feasible for on-line target localization.

## Introduction

Recently, commercial on-board cone-beam computed tomography (CBCT) system is available for image guided radiotherapy (IGRT) to provide physicians the opportunity to pinpoint the target location while setting up patient on the treatment couch before each treatment fraction.^1^–^6^ Especially for those sites with moving targets such as the prostate, knowledge of the exact position of planning target volume (PTV) would improve the output of treatment.^7^ More accurate delivery of the prescription dose to PTV would permit a tighter margin, accounting for setup error of the patient and movement of the target volume. Reducing the margin would spare more surrounding normal tissue and provide an opportunity for dose escalation on PTV.^8^–^9^ Dose escalation has been proven to be an effective way to increase the probability of disease control. In addition, by reducing the margins normal tissue complications may decrease. Therefore, it is important to investigate the localization accuracy of current image registration algorithms when varied amount of anatomical information was used, and to find a best combination of them for high-precision target localization.

An image-guided radiation therapy system (Trilogy™, Varian Medical System, Palo Alto, California) was installed in our hospital, and has been in clinical use since 2005. The system consists of Varian’s 21EX Clinac^®^ linear accelerator and an On-Board Imager. On-Board Imager is mounted on the treatment machine via robotically controlled arms which operate along three axes of motion, and perpendicular to the radiation beam direction. A full gantry rotation yields approximately 670 projection images and takes 1 min.^10^–^11^ Based on these projection images, the cone-beam CT (CBCT) can be quickly reconstructed after acquisition. For online IGRT, registering the PTV in the CBCT to their planning position in CT is possible but mainly conducted in a manual way, which is time-consuming and less robust as its accuracy may depend on operator. A number of automatic registration techniques have been investigated. Some of them are feature based methods and others are intensity based methods. The feature based methods compare points, curves, and/or surfaces in images and attempt to find the transformation that overlaps the two subjects.^12^–^14^ These methods are known to be fast and accurate, but they in general require feature extractions prior to the registration that are difficult to be accomplished in a fully automatic way. The intensity based methods however generally require no or little human interactions because of their direct use of image intensity values for comparison.^15^–^19^ However, due to the exponentially increased feature, i.e. complete information of image sets, they are usually time-consuming and less practical for on-line registration.^20^

To improve the time efficiency and accuracy of the registration algorithm, we developed a fast, automated three-dimensional (3D) gray-value registration method. It is based on the assumption that the PTV does not change shape significantly relative to its motion as a whole, and only three translations and three rotations are involved. Two features should be noted for this method. First, the correlation coefficient and mutual information were used for the similarity measurement. To reduce the total registration time, the correlation coefficient based search was first performed for a coarse registration, from which a fine registration was started based on the similarity measurement of mutual information. Second, a simplex downhill algorithm was employed to iteratively search for an optimal target to minimize the cost function. Based on the developed registration algorithm, three window-level (WL) combinations, Bony only (B), Bone + Tissue (BT), and Bone + Tissue + Air (BTA), commonly used for image enhancement were investigated. The effect of anatomical information on the accuracy and robustness of gray-value based registration algorithm was evaluated by extensive tests performed in searching for the attraction range of registration algorithm. Besides this, the effect of volume size used in registration was also investigated. The time efficiency and registration accuracy were evaluated by comparing performance of registration using different volume data containing region of interest.

## Methods and Materials

### Image acquisition

The CT of an anthropomorphic chest phantom was acquired by GE Lightspeed scanner. The reconstructed image size is 512 × 512 (pixel resolution of 0.98 mm × 0.98 mm) and slice thickness is 1.25 mm. To match the pixel resolutions of CBCT in the three dimensions, the plane resolution of planning CT images were re-sampled to 1.0 mm × 1.0 mm and slice thickness was interpolated to 1.0 mm. The X-ray projection images were acquired by an on-board imager (Varian medical system, Palt Alto, CA). Approximately 670 projection images were acquired with the full rotation of gantry (averagely 0.54°/projection) and the total acquisition takes 1 min. CBCT was then reconstructed by the typical Feldkamp algorithm.^21^ The sizes of CBCT volume are 30 cm × 40 cm × 25 cm along longitudinal, lateral, and vertical axes. The resolution in each dimension is 0.5 mm. For clinical application, such resolution is excessively high, and therefore, it is down-sampled to 1.0 mm × 1.0 mm × 1.0 mm in three dimensions for registration use.

### Image alignment

To ensure the robustness and the accuracy of measurements, first thing is to know the gold standard registration between two sets of images which was used as reference point. For the chest phantom, the radio-opaque fiducials were attached to the skin for setup purpose. The gold standard registration was established using fiducial marker (FM) based registration. These markers were manually identified on the magnified CT and CBCT slice images using ImageJ (http://rsb.info.nih.gov/ij/). For each marker, the coordinates of center was selected by multiple operators and the coordinates were averaged. The FM registration searched the registration solution by minimizing the average distance between the imaged fiducial markers on CT images and the markers on CBCT image. As the difference between coordinates of fiducial markers on CT and CBCT image sets was identified, the two sets of images can be perfectly aligned according to detected shifts. This result after shift correction represents the best matching between two sets of images, and is assumed to no shift between them.

### Image enhancement

**Figure 1:**
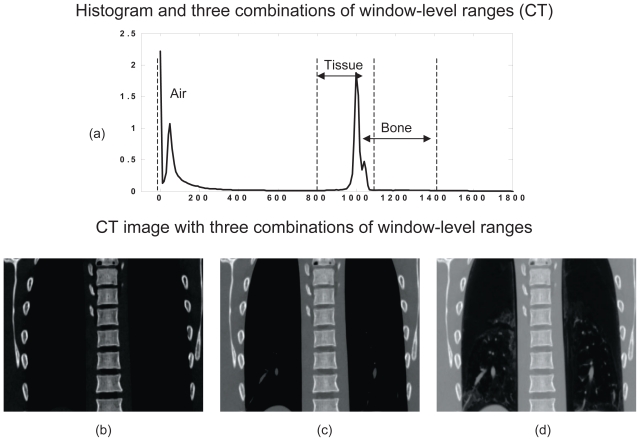
CT image enhancement. (**a**) Histogram and three sets of window-level ranges: Bone only (B), Bone + Tissue (BT), and Bone + Tissue + Air (BTA). (**b**)–(**d**) are the resulting CT images.

**Figure 2:**
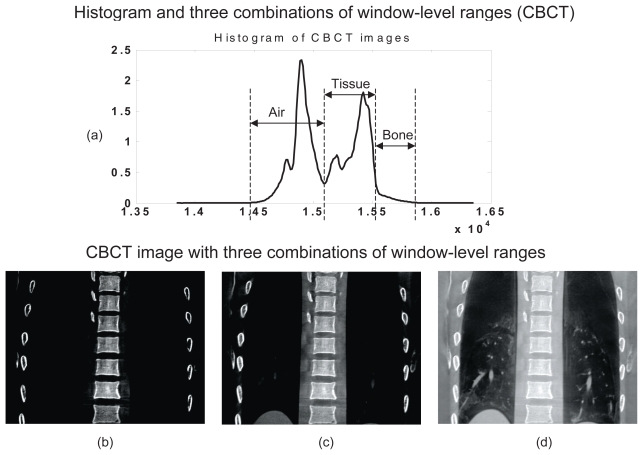
CBCT image enhancement. (**a**) Histogram and three sets of window-level ranges: Bone only (B), Bone + Tissue (BT), and Bone + Tissue + Air (BTA). (**b**)–(**d**) are the resulting CBCT images.

For image enhancement, the window-level (WL) method was used because of its computational efficiency, comparing to those of histogram equalizations (HE) and the adaptive histogram equalization (AHE) method which manipulates image in a delicate manner but has high computational cost.^20^ The window-level (WL) based image enhancement method is commonly used on medical images that linearly stretches the user selected image histogram range to the full range of the display resolution. The intensity values are calculated by the following function.

(1) $$I^{\prime }=\{\begin{array}{ll} l_{d}-w_{d}/2 & \left(I<l-w/2\right)\\ w_{d}/w(I-l)+l_{d} & \left(I>l-w/2,I<l+w/2\right)\\ l_{d}+w_{d}/2 & \left(I>l+w/2\right) \end{array}$$

where *I* and *I’* are the input and output intensity values, *w* and *l* are the user selected window and level values, and *w_d_* and *l_d_* are the display window and level parameters, which are typically 256 and 128, respectively. Three different sets of WL parameters commonly used in image enhancement method were empirically selected based on the CT and CBCT histograms. They are the window-level (WL) combinations of bone-only (B), bone + soft tissue (BT), bone + tissue + air (BTA). The selected ranges are indicated in Figure 1a for CT and in Figure 2a for CBCT. The enhanced CT and CBCT images with different WL combinations are shown in Figure 1b–Figure 1d and Figure 2b–Figure 2d. Note that the value range of histogram shown in Figure 2a might vary due to different scale coefficient applied in reconstruction algorithm.

### Similarity measurements

Two of the most popular coefficients in measuring similarity between two sets of images, the correlation coefficient (CC) and the mutual information (MI) were chosen in this application. The true correlation coefficient of two random variables *X* and *Y* is defined as,

(2) $$C(X,Y)=\frac{E(X-EX)E(Y-EY)}{\sqrt{DX}\sqrt{DY}}$$

where *E*(.) is the mathematical expectation, *D*(.) is the variance. In the image registration context, *X* and *Y* are the normalized one-dimensional vectors with corresponding elements from each image set. The large value of CC indicates the close similarity between two sets of images. The mutual information of two random variables *X* and *Y* is defined as,

(3) $$I(X,Y)=\sum_{x,y}p(X,Y){\text{log}}_{2}\frac{p(X,Y)}{p(X)p(Y)}$$

where *x* and *y* are grey levels, *p*(*X*, *Y* ) is the joint probability density function (PDF) of random variables *X* and *Y* and *p*(*X* ) and *p*(*Y* ) are the marginal PDFs. In the case of image registration, *p*(*X* ) and *p*(*Y* ) are the normalized histograms of image X and image Y and *p*(*X*, *Y* ) is the normalized joint histogram of them. Again, the large value of MI indicates close similarity between two sets of images. The similarity measure is then simply defined as the CC or MI between CT and CBCT images

(4) $$S_{C}(X,Y)=C(Image_{reference}(X),Image_{target}(Y))$$

(5) $$S_{I}(X,Y)=I(Image_{reference}(X),Image_{float}(Y))$$

where **X** = [*r_x_*, *r_y_*, *r_z_*, *t_x_*, *t_y_*, *t_z_*]^T^ and the parameters define the three rotational shifts (*r_x_*, *r_y_*, *r_z_*,) and three translational shifts (*t_x_*, *t_y_*, *t_z_*) from the origin pose. The rotation is against the 3D image isocenter, not the image origin, to prevent large displacement from small rotation angles.

### Optimization algorithm

The algorithm is intensity-based to optimize the fit between the planning CT images and CBCT images calculated as the CC and MI. The algorithm uses multiple start positions of CBCT images with respect to the gold standard registration (i.e. the CBCT images is offset from their gold standard registration by a constant shift, such as 5 mm in all the six degrees, respectively). For each start point, a simplex downhill search algorithm as explained in Appendix was performed to find the best pose to minimize the cost function. Of these six gray-value registrations, the one with the highest correlation ratio was selected. The registration algorithm consists of following basic steps.

1. Open CT images and defines the region of interests (ROI) for registration purpose. By default, the whole CT volume was used. For the fast registration, only partial volume with interested structures is used, such as the volume containing PTV plus 5 mm margin.
2. Open CBCT images and load images with the same size of volume into CPU memory. Pre-align CBCT with CT based on FM alignment vector.
3. Apply a shift Δω to CBCT to simulate daily shift of patient setup.
4. Calculate the cost function between CT and CBCT. The correlation coefficient and the mutual information were used in two searching stages with coarse and fine grids.
5. Find Δω to minimize the cost function. A simplex downhill search algorithm which progressively changes Δω to shift to the adjacent position with the lowest cost function was employed.

### Study outline

Registrations should be robust against large initial pose differences between the two registered subjects. In this study the robustness of registration algorithm was investigated by estimating the attraction range of the successful registration. Estimating the full attraction range of an optimization or a registration method in the high-dimensional search space is hardly possible, because it is difficult to visualize more than two dimensions and the number of registrations that must be performed is an exponential function of the search space dimension. Instead, we estimate a pseudo-attraction range by performing registrations with initial single-dimensional errors. The initial errors are made by shifting from a gold standard registration. A registration trial is considered successful if all elements of the error vector are less than a preset threshold, 1mm and 1 degree in this study. The attraction range is defined as the successive successful registrations in each dimension. The average error vectors of the successful registrations serve as the registration accuracy or error.

In order to investigate the possibility of on-line registration using this algorithm, the different sizes of volumes were tested. In this study, the central volume of CT and CBCT with size of 256 × 256 × 160, 256 × 256 × 80, 128 × 128 × 80, and 128 × 128 × 40 were cropped for original data set and used as ROI for registration. The three combinations of WL parameters were applied to each image set and then used for registration. All tests were performed on a computer with a 1.99 GHz CPU and 2 GB memory.

## Results

**Figure 3:**
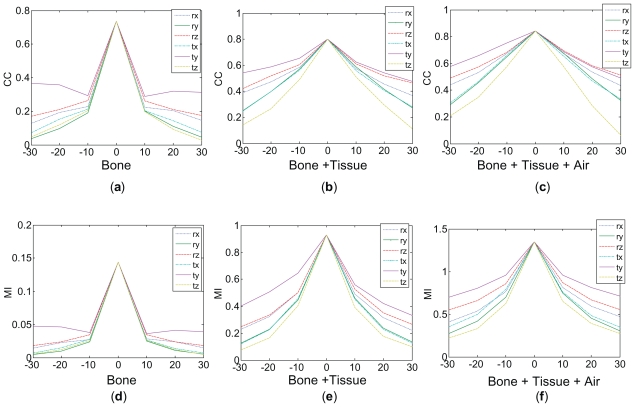
(**a**)–(**c**) CC profiles and (**d**)–(**f**) MI profiles of the three combinations of CT and CBCT enhancement methods. In each plot, three curves represent the rotation profiles and another three curves represent the translation profiles. Each profile was labeled by respective style of line. The X axes indicate the shifts in mm and deg in six dimensions, and the Y axes are the values of CC and MI.

First, CC and MI profiles for the three WL combinations are plotted in Figure 3. They were calculated by corresponding Matlab routines. The profiles (a)–(c) were generated by calculating values of CC while shifting the CT image in a single dimension relative to CBCT images from the FM based registration. The profiles (d)–(f) were generated by calculating values of MI in the same way. The shifts range from −30 to 30 with a step size of 1 mm for translations and 1 deg for rotations. The profiles of WL combination of B do not monotonically decrease the shift range beyond 10 mm and 10 degree. The profiles of WL combination of BT and BTA show good accuracy (as the peaks happen at the origin) and have smooth convergence. Profiles calculated based on CC show smaller gradient of profiles than those of MI. These single-dimension profiles provide us with valuable information for optimization method design and for the expected performance. However, the actual registration result may be different since the profiles only show a small subset of the actual underlining CC and MI similarity functions.

**Figure 4:**
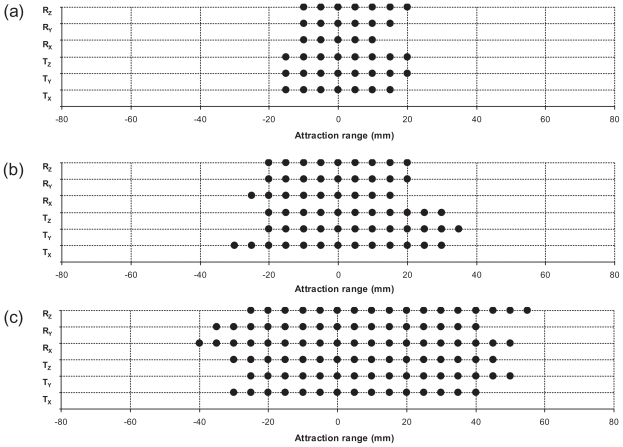
Registration grid plots for the WL combinations of (**a**) B, (**b**) BT, and (**c**) BTA. The X axes are the initial offset (mm for translations, deg for rotations), and the Y axes are the dimensions offset were made. Each symbol represents one registration trial. A dot “•” indicates that the initial offset was successfully detected, and an “ ” indicates failure of registration, i.e. the error is more than 1 mm.

**Figure 5:**
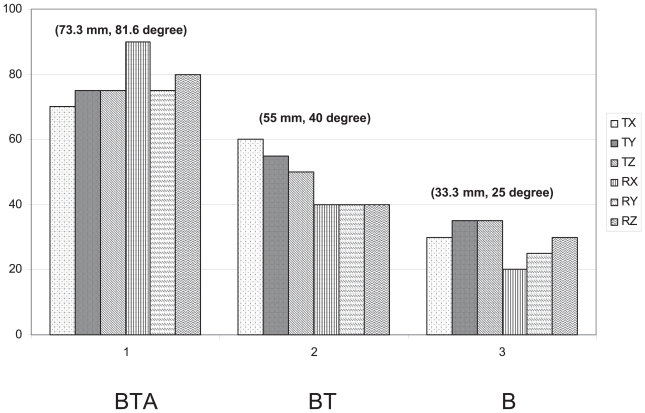
Absolute attraction ranges of the three WL combinations in six dimensions.

Full 6D registrations were conducted with initial single dimensional errors for the remaining six combinations, and the results are presented in Figures 4 and 5, and Table 1. Figure 4 shows the grid plots for the WL combinations of B, BT, and BTA, intended to show the attraction range measurements. The X axes of the plots are the initial errors in mm for translations and in degree for rotations, and the Y axes are initial offset dimensions. Each symbol on the plot corresponds to one registration trial, with a dot “•” indicating that the initial error was successfully corrected by the optimization, while an “ ” indicates that it failed. Figure 5 summarizes the attraction ranges of all six combinations. The lengths of bars represent the average rotation and translation attraction ranges for these preprocessing combinations. The numbers in parentheses are the average rotation and translation attraction ranges for each preprocessing combination. The WL combination of BTA shows the largest attraction range (73.3 mm and 81.6 deg). The WL combination of BT shows the second largest attraction range (55.0 mm and 40.0 deg). The WL combination of B gives the smallest attraction ranges (33.3 mm and 25.0 deg). Registration errors in different dimensions are summarized in Table 1. The value in each cell is the average and standard deviation of the successfully attracted registrations. All cases present subvoxel accuracy (voxel size: 1.0 × 1.0 × 1.0 mm3). On average, the translation errors are less than 0.5 mm and the rotation errors are less than 0.5 deg within attraction ranges. Among three WL combinations, BT combination presents the smallest translational errors.

For the clinical use, the registration speed is another concern since only 1–2 minutes is allowed to accomplish the whole process. The time efficiency of different registration volume size is reported in Table 2. The subvoxel accuracy was achieved for all cases examined regardless the volume sizes. Among four sets of image with different volume sizes, set A achieved the least registration errors in most of tests with three WL combinations, while Set D presented the largest registration errors in most of tests. As a trade-off, Set A took the longest time while Set D took the least time to be accomplished due to its small volume size. There is a tendency of decreasing registration time while volume size of CT/CBCT became smaller. A significant increase of registration time is observed as volume larger than 256 × 256 × 160, which is the half size of original CT/CBCT image volume.

## Discussion

First of all, The CC and MI profiles along longitudinal axis of Figure 3a and Figure 3d did not show a consistent convergence property over the full range of shifts. There is a local minimum at the shifts of −10 mm and 10 mm. The WL combination of B also did not have good MI profiles (Fig. 3d). The maximum MI values (0.14) at the origin indicate that the dissimilarities between the processed CT images and the CBCT images were large. Therefore, the WL combination of B is not expected to provide a good registration accuracy, which was confirmed by those results shown in Figure 4 and Figure 5. Therefore, the WL combination of B is not recommended for registration purpose unless the registration mask was applied. The WL combination of BT showed the wider attraction ranges (on average 55.0 mm and 40.0 deg) than those of WL combination of B due to the inclusion of soft tissue. The WL combination of BTA shows the widest attraction ranges (on average 73.3 mm and 81.6 deg) as well as good enough accuracy for patient setup (0.15 mm and 0.32 deg). Its wide attraction range might come from the fact that all anatomies were included with minimum histogram modification. Another advantage of this combination is no user interaction is required.

It is also noted that the measured six orthogonal MI profiles prior to the full registrations provided rough estimates of the full registration attraction measures. Comparing the attraction ranges shown in Figure 4 and the profiles in Figure 3, we see that there is a good agreement between them. For example, the steep curvatures of the CC profile (Fig. 3a) and the MI profiles (Fig. 3a) of the WL combination of B indicate a small attraction range of registrations (Fig. 4a). The smooth curvatures of the CC profile (Fig. 3c) and the MI profiles (Fig. 3f) of the WL combination of BTA indicate a large attraction range of registrations (Fig. 4c). The CC profiles (Fig. 3b) and MI profiles (Fig. 3e) of the WL combination of BT present similar curvatures which are less smooth than those of BTA. One may attempt to use the profiles instead of conducting full registrations, which has the benefit of fast comparison and independence from the underling optimization method and parameters.

Using typical sizes of CBCT and planning CT, the current algorithm is still not sufficient to accomplish registration within the clinical time constraint (usually within 1~2 minutes). As demonstrated in Table 2, the time cost on registration can be exponentially increased as the size of CT/CBCT volumes exceeding a certain value due to explosion of search space. To address this issue, the resolution of CT and CBCT were usually reduced by a factor of 2 or 4 in practice. As indicated in Table 2, the significant improvement of time efficiency of registration algorithm was achieved as the size of CT/CBCT volume was reduced by 2–4. Alternatively, without losing the resolution of CT/CBCT of planning target, the registration can be confined to a small region. Such an approach, as employed in this study, was popularly used by several investigators when their registration algorithms were applied in real clinical scenario, and comparable results were achieved similar to those by registering the whole volumes of CT/CBCT. It should be noted that the given results are valid only with the described method in the specified environment. The results may be varied due to different similarity measurements, regions of interest used, and image enhancement approaches adopted. However, it is clear from the experimental results that the presented registration based on the CC and MI similarity measure is affected apparently by the image enhancement methods, especially its robustness. Properly selection of the image enhancement method will significantly improve the registration accuracy and robustness.

## Conclusion

We have conducted an investigation on the registration accuracy and robotness of a rigid body based 6-degree registration method. Three WL combinations commonly used in image enhancement were examined to assess their effects on accuracy and robustness of the registration algorithm. The WL combination of BTA showed the best performance with widest attraction ranges as well as subvoxel registration accuracy. The WL combination of BT is secondary. In a conclusion, the minimal linear histogram modification on both CT and CBCT images provides the best robustness and highest registration accuracy among three WL combinations investigated. The registration efficiency was also investigated with different image volumes. It shows that significant decrease of registration time was achieved as image size reduced by half or more. It basically meets clinical time constraint for on-line target registration without compromise of registration accuracy.

## Figures and Tables

**Figure 6:**
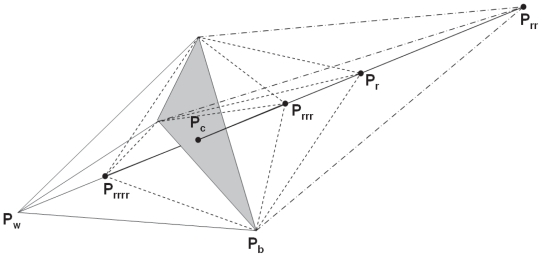
Graphic illustration of step (5) of simplex searching algorithm.

**Table 1:** Attraction range of three window-level combinations.

Dim	Registration error (mm)		
	BTA	BT	B
T_X_	−0.1 ± 0.2	0.0 ± 0.2	0.1 ± 0.2
T_Y_	0.1 ± 0.3	0.0 ± 0.3	0.1 ± 0.5
T_Z_	0.2 ± 0.3	0.0 ± 0.3	0.0 ± 0.2
R_X_	0.4 ± 0.2	−0.1 ± 0.3	0.1 ± 0.4
R_Y_	0.3 ± 0.1	0.1 ± 0.2	0.0 ± 0.2
R_Z_	0.0 ± 0.2	0.0 ± 0.1	0.1 ± 0.2

**Abbreviations:** BTA: Bone + Soft tissue + Air; BT: Bone + Soft tissue; B: Bone.

**Table 2:** Registration times with different data sets.

Dim	CT–CBCT data set in size of											
	(A) 256 × 256 × 160			(B) 256 × 256 × 80			(C) 128 × 128 × 80			(D) 128 × 128 × 40		
	
	BTA	BT	B	BTA	BT	B	BTA	BT	B	BTA	BT	B
T_X_	−0.1 ± 0.2	0.0 ± 0.2	0.1 ± 0.2	0.1 ± 0.1	0.0 ± 0.1	0.0 ± 0.1	0.0 ± 0.1	0.0 ± 0.1	−0.6 ± 1.2	0.1 ± 0.2	0.0 ± 0.2	0.5 ± 1.3
T_Y_	0.1 ± 0.3	0.0 ± 0.3	0.1 ± 0.5	−0.4 ± 0.2	−0.1 ± 0.2	0.1 ± 0.2	−0.4 ± 0.2	−0.1 ± 0.3	−0.1 ± 0.7	0.3 ± 0.2	0.4 ± 0.2	−0.5 ± 1.2
T_Z_	0.2 ± 0.3	0.0 ± 0.3	0.0 ± 0.2	0.0 ± 0.1	0.1 ± 0.1	0.1 ± 0.1	−0.1 ± 0.1	0.0 ± 0.1	0.1 ± 0.2	0.0 ± 0.1	0.2 ± 0.1	0.0 ± 0.4
R_X_	0.4 ± 0.2	−0.1 ± 0.3	0.1 ± 0.4	−0.6 ± 0.3	−0.1 ± 0.1	0.1 ± 0.1	−0.6 ± 0.2	−0.2 ± 0.2	−0.1 ± 0.6	0.5 ± 0.2	0.1 ± 0.2	−0.6 ± 1.3
R_Y_	0.3 ± 0.1	0.1 ± 0.2	0.0 ± 0.2	−0.3 ± 0.2	0.0 ± 0.1	0.0 ± 0.0	−0.3 ± 0.1	0.0 ± 0.1	−0.5 ± 1.2	0.1 ± 0.2	−0.4 ± 0.2	0.3 ± 1.2
R_Z_	0.0 ± 0.2	0.0 ± 0.1	0.1 ± 0.2	−0.1 ± 0.1	−0.1 ± 0.1	0.0 ± 0.0	−0.1 ± 0.2	0.1 ± 0.2	0.2 ± 0.4	−0.1 ± 0.2	0.4 ± 0.9	−0.8 ± 0.8
Ave Time	18 min			6.2 min			2.4 min			1.5 min		

**Abbreviations:** BTA: Bone + Soft tissue + Air; BT: Bone + Soft tissue; B: Bone.

## Appendix I The approach of downhill (Nelder-Mead) simplex optimization

The downhill simplex method is initially proposed by Spendley, Hext, and Himsworth (1962), and then improved by Nelder and Mead (1965). It is a linear fitting procedure to mathematical functions, which may be applied to non-linear problems. The term “simplex” arises because the feasible solutions for the parameters may be represented by a polytope figure called a “simplex”. The simplex for the case of a function of vector in *N* dimensions is stored as a *(N* + *1)* × *N* array. Each column of the array contains *N* elements and represents a vertex of the polytope. In the 2-dimensional case the simplex is a triangle, and in 3-dimensional case it is a tetrahedron. The algorithm is given an initial set of vectors in the simplex and proceeds to find the function minimum by a process of reflection expansion and contraction of the simplex. The algorithm invariably converges to a minimum following a series of contractions so that the final simplex contains very similar vectors in each column. The Simplex method differs from the widely used linear optimization algorithms in that it does not use derivatives, which confers safer convergence properties to the Simplex method since it is much less prone to finding false minima. The basic procedure consists of six steps as follows:

1. Defining an initial simplex with initial vector **P**_0_ and the other six vertex vectors **P**_i_ = **P**_0_ + a_i_**e**_i_, (i = 1, …, N = 6) where **e**_i_ are unit vectors and a_i_ are constants that characterize the length for each vector direction.
2. Evaluating S(**P**i), i = 0, …, N, based on Eq. (4) or (5), and sorting them in descending order. Locating the best, 2nd worst, and worst vectors in the sorted list and labeled as vectors **P**_b_, **P**_g_, and **P**_w_, respectively.
3. Calculating the centroid of the first six best vectors and defines it as $P_{c}=\frac{1}{N}\sum_{i=1}^{N}P_{i}$.
4. Calculating the reflection point of the worst vector by **P**_r_ **= P**_c_. + α**(P**_c_ **− P**_w_), where **P**_r_ is point on another side of simplex opposite to the **P**_w_.
5. Updating **P**_w_ with a candidate vector for achieving the best improvement of function. The following rules are used and illustrated in figure below:
  If S(**P**_r_) >S(**P**_b_), then **P**_w_ = **P**_rr_ = **P**_c_ + β(**P**_c_ **− P**_w_);
  Else if S(**P**_r_) >S(**P**_g_) & S(**P**_r_) ≤S(**P**_b_), then **P**_w_ = **P**_r_;
  Else if S(**P**_r_) >S(**P**_w_) & S(**P**_r_) ≤S(**P**_g_), then **P**_w_ = **P**_rrr_ = **P**_c_ + γ (**P**_c_ **− P**_w_);
  Else **P**_w_ = **P**_rrrr_ = **P**_c_ **−** γ (**P**_c_ **− P**_w_);
6. Determining the convergence of procedure. Calculating $\bar{P}=\frac{1}{N}\sum_{i=1}^{N}P_{i}$ with **P**_w_ updated. If $\frac{1}{N}\sum_{i=0}^{N}||P_{i}-P||\;<ɛ$ or the maximum number of iteration *N**_max_* was arrived, the searching stopped and *P̄* is the vector for final output. Otherwise, the searching will continue by returning to step (2).

By default, for a better compromise between efficiency and effectiveness of this program, the following parameters, α, β, γ, ɛ, *N**_max_*, have to be adjusted. In this case, they are set by α = 1, β = 2, γ = 0.5, ɛ = 0.05, *N**_max_* = 50.
